# The role of philosophy of science in Responsible Research and Innovation (RRI): the case of nanomedicine

**DOI:** 10.1186/s40504-014-0005-8

**Published:** 2014-04-26

**Authors:** Gry Oftedal

**Affiliations:** Department of Philosophy, Classics, History of Art and Ideas, University of Oslo, Box 1020 Blindern, 0315 Oslo, Norway

**Keywords:** Causation, ELSA, Ethics, Nanomedicine, Philosophy of science, Reduction, Root cause, RRI

## Abstract

Research on ethical, legal and social aspects (ELSA) of life sciences and new technologies has mainly been focused on impacts and consequences, while the emerging framework of Responsible Research and Innovation (RRI) focuses rather on increased involvement and reflexivity in research processes to foster science and technology that better answers the needs of society. I argue that philosophy of science should be a central feature of RRI and demonstrate how the philosophy of science can contribute in this sense. I show how investigating basic assumptions in research, here exemplified by reductive assumptions in causal modeling, can have important ethical and societal implications.

## Introduction

Responsible Research and Innovation (RRI) is a framework for European research in new and emerging areas of science and technology that has been proposed as an alternative to the ELSA mode of addressing Ethical, Legal and Societal Aspects of new scientific developments. While ELSA mainly takes issues with various impacts of science on society, the RRI framework places more weight on the *process* of research and innovation, aiming at larger involvement and reflexivity while emphasizing openness, transparency and dialogue (Sutcliffe 2011; European Commission 2012; Von Schomberg 2013). It also comprises a move from the mainly external and theoretical approaches of the ELSA framework to more integrated projects, in which aspects of RRI will be incorporated into science research projects. A suggestion is that science conducted within an RRI framework will meet the interests and goals of society more effectively by opening up the process.

Some central RRI values have been suggested and discussed. However, what is the more specific content of RRI is largely left open. Some will for this reason deem the concept too vague, but giving an exact definition of RRI is not necessarily fruitful. Leaving it open for particular projects to define RRI relative to their research context and in cooperation with various stakeholders, is one way to create discussions particularly relevant to the projects at hand. Taking this perspective, how RRI is implemented in current and future research will and should vary significantly from project to project.

RRI approaches still need some common ground, and I want here to make the case for establishing philosophy of science research as a central feature of RRI, not least because openness, transparency, and a broader involvement in research and innovation will require methods, assumptions, and values in research to be explicit, understood, and discussed. An RRI perspective will allow for asking foundational questions regarding methods and scientific assumptions, and philosophy of science comes into view more easily compared to ELSA approaches. This paper is intended as a first demonstration of what philosophy of science research has to offer an RRI framework for nanomedicine with a focus on reductionist assumptions and choices in causal modeling.

There are numerous definitions of ‘nanomedicine’, one of the most frequent being ‘the application of nanotechnology to health’ (European Commission 2005, 2006). European policy documents describe nanotechnology as ‘the study of phenomena and fine-tuning of materials at atomic, molecular and macromolecular scales, where properties differ significantly from those at a larger scale’ (European Commission 2013). Nanomedicine has become an interdisciplinary subject area and is surrounded by huge expectations. The European research strategy focuses on developing the use of nanotechnology in targeted drug delivery, diagnostic *in vivo* imaging, and in regenerative medicine (European Commission 2006, 2009). It is also a goal to make nanomedicine more personalized as well as more cost-effective than other approaches. Current nanotechnological research enables novel applications such as imaging, measuring, modeling, and manipulation of nano-sized matter. It helps to reveal mechanisms of disease, to refine molecular diagnostics, and to discover, develop, and deliver drugs (e.g. Hrkach et al. 2012).

Recent ELSA research on issues related to nanomedicine is focused mainly on the risk of undesired impacts. Risk and safety are major concerns, such as possible health risks due to toxicity both when nanomaterials are used in the human body and when exposed to the environment (Resnik and Tinkle 2007; Manchikanti and Bandopadhyay 2010). Regulative issues (e.g. Guerra 2008) are also debated, as is the question of access to health care and a possible economic nano-divide between those with access to the technology and those without (Hodge et al. 2007; Maclurcan 2009). In other discussions, scholars take issue with personalized medicine (e.g. Pellé and Nurock 2012) and ethical aspects of nanomedicine in clinical trials (e.g. Berger et al. 2008). There is also the question of the use of nanotechnology for human enhancement (e.g. Hassoun 2008; Ferrari et al. 2012). While these are important discussions, few have so far exposed basic and implicit assumptions of nanomedical research.

According to EGE opinion no. 21 (European Commission 2007, 60), more research and a deeper understanding should be promoted concerning ‘the broader questions of nanomedicine, among other things individual responsibility, including the shifts in the concept of the self, personal identity, societal goals, and global health care’. There are foundational aspects of nanomedicine with potentially important implications for these questions. As recognized by Khushf (2007), ethical analysis should also consider some of the reductionist assumptions in models integral to nanomedicine to keep the broader perspectives of nanomedicine under public scrutiny.

Studies of social and societal aspects of science, often labelled Science and Technology Studies (STS), include discussions of the reductive aspects of emerging sciences (e.g. Wynne 2005). Important contributions come from this tradition; however, I will in this paper press the philosophy of science viewpoint, analyzing scientific activity from within and looking at the specific processes of modeling and explanation. What I am suggesting is that analyzing foundational issues related to emerging sciences from a philosophy of science perspective remains of great relevance to societal, ethical, and policy questions in a manner that is undervalued.

I will address some of the assumptions and basic views related to reduction and causation in nanomedical research and suggest how they have significant ethical relevance. I especially address the assumption that ‘root causes’ of diseases are to be found at the molecular level. I also indicate the manner in which the call for more philosophy of science fits in to the transition from ELSA to RRI in European biotech research.

## Reduction and systems approaches in biology, medicine, and nanomedicine

In the philosophical literature, reduction comes in many variations including ontological, methodological, and epistemic reduction. Discussions among philosophers of science has long mainly concerned the relation between scientific theories debating for instance the questions whether classical genetics theory and concepts can be reduced to molecular genetics theory and concepts (Schaffner 1967; Hull 1974). More recently, however, this approach to reduction in the biological sciences has been deemed less fruitful, and there is now more focus on the role of reduction in explanation (Waters 1990; Bickle 2003) and its relation to accounts of causation (Dupre 1993; Strand and Oftedal 2009).

The modes of reduction that have been especially prominent and very successful in many biological approaches are methodological and epistemic reduction. The most widespread way to gain knowledge about living systems has been to break them into parts and look at them individually and in interaction with one or a smaller selection of other parts. Such an approach may also bring with it explanatory reduction, that is, the process of explaining the workings of living systems from the workings of (some of) its parts. In this paper I use the term reduction in an epistemic-explanatory way and refer to the following aspects. Reduction involves (1) investigating parts and mechanisms in order to understand a system in a bottom-up manner; (2) explaining an effect by only selected causes and/or only at some specific level/scale of interest; (3) ascribing causal responsibility mainly to lower levels, typically the molecular level; and/or (4) assuming the independence of composite mechanisms (=modularity assumptions). Often several or all of these aspects are involved in biomedical/nanomedical research (e.g. Kim et al. 2010).

Reductive approaches are by no means exclusive to nanomedicine, although there are several aspects of nanomedicine differing from molecular genetics and other biological research approaches in this respect. These differences include the possibility of conducting extremely precise interventions at nano-scale levels. There are also new applications such as real-time detection and monitoring technologies, where researchers are no longer dependent on traditional intervention experiments, but can follow molecular processes in real time (e.g. Berthing et al. 2011; Panikkanvalappil et al. 2013). These possibilities are already in play and may open numerous molecular black boxes. In summary, new applications in nanomedicine turn reduction into something more concrete compared to previous approaches. We can now, and probably even more in the future, change and influence matter atom by atom, or molecule by molecule. This brings new dimensions to reduction in science.

Sometimes reduction is portrayed as inherently negative and part of a naïve realist/reductionist scientific worldview (e.g. Wynne 2005). It is more fruitful, I hold, to view reduction as a scientific tool needed in research. Scientists for the most seem to acknowledge that their models give a simplified picture of a slice of reality in which certain features are accented and others ignored. Such a view of scientific modeling is comparable to R. Giere’s (2006) perspectival realism. Focusing on certain aspects is needed to be able to examine the portion of the world under investigation. Importantly, however, choices in modeling affect what causes of a phenomenon of interest are revealed and accentuated (assuming a phenomenon typically has many causes). It should be far better communicated that the causes under investigations are only a small selection from a complex network of causal factors. Many aspects are left in the fringes or out of the picture. These choices should be made more explicit, discussed, and understood. If scientists are not careful, conscious, and communicative about dealing with simplifications and partial explanations, it could result first in a biased understanding of causal relations and second in biased policies regarding the implementation of interventions such as those affecting people’s health.

Systems approaches have recently enabled researchers to focus on larger and larger portions of the world simultaneously. These methods demand more and more complex modeling – often at the cost of manageability and comprehensibility. Still, even complex models need to focus on a limited number of aspects and features of the world. For instance, many systems biology models based on large amounts of data depict network properties (e.g. connectivity) in a defined system rather than detailing molecular mechanisms (e.g. van der Greef et al. 2007).

Ahn et al. 2006 present a timely analysis of reduction in medicine. The pervading paradigm in medicine is, they suggest, reductionism in the sense that we typically focus on one factor as a disease cause or target for disease intervention (corresponding to aspect number 2 defined above). We typically also treat diseases in an additive manner; we treat each disease in a disease complex as an independent phenomenon instead of addressing connections and interactions between different pathologies in the same individual (corresponding to aspect 4 defined above). Additionally, models in established medicine are typically linear, predictable, and frequently deterministic; health is seen as normalcy, risk reduction, and homeostasis. As an alternative or complementary approach, Ahn et al. 2006 propose the systems perspective used in current systems biology. Many systems approaches focus on several causal factors simultaneously and pay attention to context and complex interactions. Models are typically non-linear and sensitive to initial conditions, and health is viewed as robustness and adaptation/plasticity.

Nanomedical research often involves systems approaches where a large amount of factors are considered simultaneously and where the focus is on systemic output of nano-intervention and knowledge of interactions between many system components (see Bradbury et al. 2008). But even though systems biologists and nanomedical researchers may claim they apply non-reductionist approaches (see Boogerd et al. 2007), there are important ways in which many current systems approaches are still reductionist. And while context is allowed to play a more prominent role and more factors are assessed simultaneously in systems approaches, they are typically only internal factors (cell-internal and body-internal), and typically targeted through interventions in specific molecular pathways (corresponding to aspects 1 and 3 above). Thus the molecular level is salient and even more important in systems-oriented medicine than in past medical research. Researchers are still taking complex systems apart and explaining from lower levels, the difference is that they can juggle more parts and more interactions between parts at the same time. Nanomedicine can therefore be viewed as both a more reductive option (more focus on lower-level causes and mechanisms) and a less reductive option (more use of systems approaches taking into account many factors simultaneously and allow for more complex modeling of disease interactions). Although many current systems biology approaches are reductionist in the sense discussed above, there are schools in the life sciences that are non-reductionist in the sense that they address higher- level principles rather than lower-level mechanisms (see e.g. Wolkenhauer and Green 2013).

Finally, a way in which nanomedicine is reductionist (corresponding to aspect 2 above), similar to most research in the life sciences, is that it does not take into account aspects of human beings such as personhood, feeling of self, free will and responsibility. Questions of health and disease are typically strongly connected to our self-image: who we are, how we cope, how we act in the world. There is a danger that such aspects, and other factors, such as environmental, social and psychosocial conditions, may be even more suppressed in future medicine. The possibility of precise molecular diagnosis and treatment excites researchers and funding bodies. Medicine has for a long time had a strong molecular perspective, but with nanomedicine, health and disease could be moved to the molecular scale to an even larger degree than before. In the next section, I elaborate on why this is not justified.

There is, however, an important complication in the discussion of nanomedicine which may preclude the understanding of more foundational assumptions. Two sides of nanomedicine clash in their portrayal and understanding of the science, and one is more reductionist than the other. On the one hand, there is the clinical, practical and incremental side to nanomedicine where the technology is seen as one of tools to solve clinical problems. In this approach, there is believed to be continuity in the development of medical research and practice where research approaches and ethical issues of nanomedicine can be expected to be similar to previous and parallel approaches. Researchers that find themselves well placed in this more pragmatic research focus on nanomedicine have problems seeing anything particularly new about nanomedicine or possible ethical concerns of nanomedical research (e.g. Kuiken 2010). Costa et al. 2011 found that many scientists reflect with ambiguity on the reputed novelty of nanomedicine and what the ethical issues and risks are in their work. Researchers often see no need for a shift in ethical considerations, but view ethical issues in nanomedicine as overlapping with those of other areas of biomedical research (Wickson et al. 2008).

On the other hand, there is a more visionary side to nanomedicine which is promoting a whole new approach to medicine as a practice (Leontis and Agich 2010). This side includes more speculative science writers like R. Freitas (1999), where we find nanomedicine portrayed as involving highly reductionist representations of biological systems and of human health and disease. According to Freitas (1999), “nanomedicine phenomenologically regards the human body as an intricately structured machine with trillions of complex, interacting parts, with each part subject to individual scrutiny, repair, and possibly replacement by artificial technological means”. The idea is that when we have produced molecular-level descriptions of a system, we can directly apply nanotechnological engineering principles to prevent, diagnose and treat at the molecular level (Khushf 2007, 518).

Many researchers consider these descriptions of nanomedicine to be fairly remote from everyday pragmatic research and say little of the work and assumptions of the actual working scientists (see Wickson et al. 2008). However, the line between science and science fiction is effectively blurred also by researchers and policy makers using similar formulations. In the initial American NIH Nanomedicine Roadmap Initiative disease is sometimes referred to as ‘a broken part of a cell’, and a long-term goal for nanomedicine is partly defined as being able to replace such a broken part ‘with a miniature biological machine’ (Tibbals 2011, 52). Although European reports tend to be more modest, there are nevertheless formulations expressing a single-cell understanding of disease in nanomedicine: “In nanodiagnostics, the ultimate goal is to identify disease at the earliest stage possible, ideally at the level of a single cell” (European Commission 2005). Discussions of reduction in nanomedicine must be careful not to confuse the more speculative visions with assumptions in actual research and applications. A useful philosophy of science treatment of reduction in nanomedicine I think should mainly concern the latter, and some effort will be needed to separate them.

## Causation and levels

A worry expressed by Khushf (2008) is the inability of a biomedical disease concept based on reduction and molecular-level causation to capture the complexities of disease and reality of clinical medicine. According to Khushf, the ‘root cause’ of disease is often considered as a “dysfunction of a tissue because of pathology on a cellular or subcellular level” (quoted from Kelly 2006, 1026). “The clinical problems that physicians address involve failures of function at relatively high system levels, and these are traced to failures in tissue function, which, in turn, arise from cellular and subcellular processes. If we want a properly scientific understanding of disease, we thus need to trace things all the way down” (Khushf 2008, 434). There is an assumption that disease is caused by some underlying defect at a subsystem level, or that disease somehow *originates* from lower levels.

A view of complex causation that is close to modern research approaches and that I will use as a basis for the discussion, is that any biological state, condition, or pathology should be considered to be caused by a larger causal complex, each causal factor of which can be defined counterfactually as something that would have made a difference to the effect in question if it had been changed through an intervention (Woodward 2003).^a^ Biomedical researchers typically choose a research focus on certain causes or causal pathways because intervening in these pathways is a feasible way of influencing disease conditions given the research context at hand. The chosen causal factors are to be considered as objective causes of the condition in question as long as they are difference-makers, but as discussed in the following, they are not necessarily root causes.

Biological levels are often defined as levels of composition or organization, where higher levels are composed by lower-level constituents (Oppenheim and Putnam 1958; Wimsatt 1994). In this paper I refer to a compositional understanding of levels, however, it should be kept in mind that biological systems do not fit neatly into this description of level relations. Although molecules constitute cell components, which constitute cells, which constitute tissues, which constitute organisms, most levels are composed by components from several other levels. For instance tissues are not merely composed by cells but also extracellular matrix and free-floating molecules and ions. In nanoscience the concept of scale is often invoked replacing much level-talk. The scale concept is typically used without referring to composition, but rather indicates the size-range under focus (see e.g. Berger et al. 2008).

It is commonly assumed that when something happens at a higher level (e.g. a change in some tissue functionality, say the beating of the heart), something also happens at the molecular level. There cannot be change in higher level properties without changes at lower levels (what philosophers like to call the supervenience thesis) (Davidson 1970; Kim 1993). While this supervenience thesis is not accepted by everybody, I will assume that most researchers in nanomedicine and molecular biology do. But even if we do accept it, it is important to notice what does *not* follow from it. It does not follow that even if changes do take place at the molecular level that the main cause of an effect is also at the molecular level. Causes can be *mediated* through molecular mechanisms, still leaving the major difference-making causes larger than molecules (see e.g. Craver and Bechtel 2007). For instance eating a specific culturally influenced diet (higher level cause) can affect your health in significant ways mediated through specific molecular pathways in the body. Breaking up with your boyfriend (higher level cause) may induce a depression causally mediated by certain brain molecular activity. Medical research may produce knowledge and drugs making it possible to intervene directly in the mediating molecular pathways and thereby restore health partially or fully. Say that we are able to change a pathological process by intervention at the molecular level. Even though this is possible, it is important that it does *not* follow from this that the molecular cause(s) on which the drug or treatment in question has intervened is the main cause or ‘root cause’ of the condition in the first place.

An example from nanomedicine research concerning the treatment of obesity illustrates this (Kajimoto et al. 2013). The drug Cytochrome C was delivered through a nanosystem causing vascular changes in cells ultimately reducing body-weight in mice with a high-fat diet. Although the intuitive root cause in this case is external (diet), the obesity is still mediated by molecular mechanisms that can be intervened on, and obesity may therefore be remedied without changing the diet.

One can of course ask what qualifies a certain diet as a ‘root cause’ and not some molecular process or property as long as we take diseases typically to be caused by a causal complex rather than single and simple causes. ‘Root cause’ is not a well-defined concept, and I will suggest describing it in relation to what has been the normal causal context for a longer period of time. Often we may have an intuition about what is the root cause of a phenomenon. The following is a simplified example, for the case of the argument. While genetic variants affecting people’s likelihood of becoming obese have been there for a long time, the frequent occurrence of a certain type of diet combined with low activity levels is a newer phenomenon. Since it is the diet that has changed and not the genetic variation, the diet can be considered the ‘root cause’ of increased obesity. On the other hand, if our diet had been constant over a longer period (e.g. several generations), genetic variation should rather have been considered ‘root cause’ of variation in obesity. The concept of ‘root cause’ is here understood as a relational concept rather than as referring to an objective main cause of a phenomenon.

Although biomedical research often seeks to understand biological mechanisms and the molecular causes of disease, in practice researchers address several levels of organization simultaneously. For instance, in emerging nanomedical cancer therapies, tumor tissue and cancerous cells are targeted or reached through intervening in specific biological mechanisms, for instance through targeting specific cell receptors (e.g. Ashley et al. 2011). Several relevant levels are in play in this research: molecules, transport systems in cells, cells, tissues and organism. It is an interesting question how the relations between relevant levels in this research should be described and the roles they play in models and explanations investigated.

Khushf (2008, 432) asks for a richer account of top-down causation in our understanding of disease. The question of top-down causation is extensive and has been a central topic in philosophy for some time (e.g. Craver and Bechtel 2007; Auletta et al. 2008). Some see top-down causation as mysterious and non-scientific, since in science one hardly finds any higher-level phenomena that are not dependent on what is going on at lower levels. There are still accounts of top-down causation which do not appeal to such mysterious forces and do not really challenge the supervenience thesis. One such account is to consider top-down causation as constraints on the working of the parts coming from higher-level organizational principles or boundary conditions (van Gulick 1995; Emmeche et al. 2000). The fact that molecules are organized in different ways and shaped in different structures puts important constraints on causal relations. Another species of top-down causation that has been suggested is the effect of external/environmental causes on organisms (Craver and Bechtel 2007). Some will still argue that what is called top-down causation always acts via the molecular level, even though it is better described or identified by a higher-level vocabulary, in which case no *real* top-down causation is going on. I will not try to resolve these issues here. Suffice it to say that the concept of levels in living systems ties in with how we view health and disease, and is a question that needs more attention.

Craver and Bechtel (2007) hold that what is sometimes mistakenly considered to be top-down or bottom-up causation really are not causal relations at all, but rather constitutional relations. The main reasoning behind this is that something cannot be a part of a whole and a cause of that whole at the same time. A whole is not caused by its parts; rather, it is *constituted* by its parts. Taking this reasoning on board, a lot of what people have discussed as top-down causation is rather constitutional relations. A more thorough discussion of the implications of this view for concepts of disease and health should be investigated. A richer account of top-down causation (or constitutional relations) is definitely needed, and importantly, body-external causes and causal contexts need to be acknowledged to a greater degree both by researchers and in research communication to avoid fostering an overly simplified understanding of disease causation.

As pointed to, there is a relationship between the aspects of causation and levels discussed here with reduction as defined in the previous section. Holding root causes mainly to reside on the molecular level corresponds to reduction as described in the three first aspects: It involves an approach in which parts are investigated to understand a system in a bottom-up manner; it is an attempt to explain an effect by selected causes at one main level; and it is an ascription of causal responsibility mainly to lower levels. The discussion of top-down causation and constitutional relations connects to the possibility of ascribing causal responsibility to higher-level causes and how this can be conceptualized.

## The relevance of philosophy of science perspectives

Science is very often about surprises. Researchers find something they did not expect at all or latch onto an entirely different research path because what they investigate just behaves completely differently than originally hypothesized. Although research results often reflect specific interests and are definitely parts of social and historical context and also need to be evaluated in this light, there is also a way in which natural science has a claim to objectivity. No matter how strong your interests and biases, they do not decide how the world behaves when you interact with it, even though they may invite of certain interpretations of the behavior.

In nanomedicine choices have been made to interact with the world with a focus on certain questions, certain levels, certain forms of explanation and types of intervention. This focus seems extraordinarily fruitful and successful; it creates knowledge about biological mechanisms and how to develop new and significant applications in medicine. Nevertheless, it is still important to keep in mind that this is one type of focus with an interest in certain causes. A significant problem with the contemporary tendency towards reduction and privileging of certain causal models is that other potentially fruitful approaches, such as the investigation of higher-level causes (e.g. psychological, sociological, and environmental causes) and some low-tech preventative approaches (e.g. diet) remain under-investigated. Nanomedicine may well continue as it does and give wonderful results. It is still important for funding bodies to remain vigilant to ensure this research does not absorb resources at the expense of investigations into other highly relevant aspects of disease. Given the magnitude of market interest in nanomedicine, state funding bodies have an even greater responsibility to ensure a better balance between approaches. Current medical research could also benefit from other perspectives and incorporate external factors and combine factors in multilevel approaches.

One worry is that the framework of nanomedicine will push medicine in a specific direction where most pathologies, when it comes down to it, should be addressed at the molecular level, and where the development of treatments that fight molecular causes of disease is generally seen as the most effective solution to health problems. This attitude would also be beneficial to and expected to be promoted by drug companies. It *could* also be the most effective approach to health issues, but it is far from self-evident, and we need conceptual tools to be able to discuss these matters.

It is of great interest to understand reductionist assumptions of significant scientific approaches and how they influence our understanding and conceptualization of higher-level phenomena. What is lost in reductive approaches? The understanding of how concepts of disease and health are influenced by nanomedical reduction is of ethical concern. As sketched in this paper, a first lesson from philosophy of science is not to lose sight of external, environmental, higher-level causes of disease and be aware of the possibility of people getting more estranged from their health and their bodies (the disease is in the molecules), which may also affect feelings of responsibility in relation to own health. I would also point to the importance of communicating better the complexities of disease causation so as not to foster a simplistic and wrong approach to how we understand ourselves as biological beings as well as persons in sickness and in health.

## Widening the scope of RRI and ELSA research

In the ELSA/RRI literature on nanoscience and nanotechnology, several authors have called for a philosophy of science component able to address issues of foundational assumptions of nanoscience; such a component should be integrated into a more comprehensive approach to addressing nanoscience in society (e.g. Khushf 2007; Grunwald 2011).

Responsibility involves both moral and epistemic dimensions (e.g. Grunwald 2011, 12). There is a moral dimension to how an action can be deemed responsible or irresponsible, and there is an epistemic dimension related to the quality of knowledge of the subject of responsibility, which in this context is scientific knowledge. The quality of knowledge is connected to the quality of methods, approaches, and framework of research, in which reductive assumptions and causal modeling are significant components. Questions of how we can acquire scientific knowledge, what status this knowledge has, and what assumptions are shaping the approaches taken, are classical epistemology and philosophy of science questions that I hold will prove important in an RRI perspective.

There is a conceptual shift in European research as RRI partly takes over from ELSA as a framework for addressing important concerns regarding the effects of new technologies on society and environment. Although the concept of RRI is not yet fully developed, it has been introduced in part to facilitate a better integration of ethical, societal, and risk aspects into on-going biotech research and to promote interdisciplinary cooperation and exchange among practitioners in the natural sciences, social sciences, and the humanities. A practical implication is that research into ethical and social aspects is supposed to be funded through the biotech projects, not as independent projects, creating closer dependencies between scientists and RRI/ELSA researchers. This may result in closer collaboration, but may also promote more ‘tailor-made’ RRI research to fit the relevant projects.

Research under the ELSA framework has not had much focus on philosophy of science issues, but such research could easily be incorporated into ELSA to a greater extent. And as discussed in the beginning of this paper, the RRI framework seems, considering some of its visions and values so far, very promising when it comes to integrating more philosophy of science research. On the other hand, the expected close integration of RRI with ongoing research projects may provide some challenges for more foundational investigations of scientific concepts and assumptions. One reason is that this work may be of more general relevance than of direct relevance to specific projects. Still, if funding bodies take into account the importance of investigating philosophy of science issues in an RRI framework, and philosophers focus at making their work more directly interesting to current research, it is still feasible to integrate philosophy of science questions into on-going projects.

## Endnote

^a^One problem of counterfactual definitions of disease causation is that biological systems typically are robust and may compensate for the disturbance/intervention on many factors in the system in a way that makes these factors not appear as causes since the effect seems not to be counterfactually dependent on them. Strand and Oftedal 2013 present one possible solution to this problem for counterfactual accounts of causation.
